# Amyloid-Related Imaging Abnormalities With Anti-amyloid Antibodies for the Treatment of Dementia Due to Alzheimer's Disease

**DOI:** 10.3389/fneur.2022.862369

**Published:** 2022-03-23

**Authors:** Charles G. Withington, R. Scott Turner

**Affiliations:** Department of Neurology, Georgetown University, Washington, DC, United States

**Keywords:** Alzheimer's disease, amyloid related imaging abnormalities, aducanumab, ARIA-H, ARIA-E

## Abstract

Second-generation anti-amyloid monoclonal antibodies are emerging as a viable therapeutic option for individuals with prodromal and mild dementia due to Alzheimer's disease (AD). Passive immunotherapy with aducanumab (Aduhelm), lecanemab, donanemab, and gantenerumab all lower CNS amyloid (Aβ) burden but come with a significant risk of amyloid-related imaging abnormality (ARIA)—the most common side effect of this class of drugs. While usually asymptomatic and detected only on brain MRI, ARIA may lead to new signs and symptoms including headache, worsening confusion, dizziness, visual disturbances, nausea, and seizures. In addition, one fatality related to ARIA-E (edema) with aducanumab and one fatality due to ARIA-H (hemorrhage) with donanemab are reported to date. ARIA-E may be associated with excessive neuroinflammation and saturation of perivascular clearance pathways, while ARIA-H may be related to vascular amyloid clearance with weakening and rupture of small blood vessels. The risk of ARIA-E is higher at treatment initiation, in ApoE4 carriers, with higher dosage, and with >4 of microhemorrhages on a baseline MRI. The risk of ARIA-H increases with age and cerebrovascular disease. Dose titration mitigates the risk of ARIA, and contraindications include individuals with >4 microhemorrhages and those prescribed anti-platelet or anti-coagulant drugs. A brain MRI is required before aducanumab is initiated, before each scheduled dose escalation, and with any new neurologic sign or symptom. Management of ARIA ranges from continued antibody treatment with monthly MRI monitoring for asymptomatic individuals to temporary or permanent suspension for symptomatic individuals or those with moderate to severe ARIA on MRI. Controlled studies regarding prevention and treatment of ARIA are lacking, but anecdotal evidence suggests that a pulse of intravenous corticosteroids may be of benefit, as well as a course of anticonvulsant for seizures.

## Introduction

CASE REPORT: A 68-year-old male attorney developed gradual and progressive cognitive and functional decline and was diagnosed with mild dementia due to Alzheimer's disease (AD). He was forced to retire earlier than planned. His mini-mental state examination (MMSE) score was 24/30. He was otherwise healthy and did not require anti-platelet or anti-coagulant drugs. His brain MRI revealed diffuse atrophy and periventricular white matter changes consistent with age (non-diagnostic); a quantitative analysis was not performed. In addition, two microhemorrhages and one area of superficial siderosis were noted, but no cerebral infarcts, including lacunar infarcts, were apparent. A cerebrospinal fluid analysis of amyloid, total tau, and phospho-tau181 levels supported a clinical diagnosis of dementia due to AD and was consistent with CNS amyloid accumulation (A+/T+/N+ in the AD biomarker classification scheme). An amyloid PET scan of the brain was not obtained. His ApoE genotype, initially discovered by direct-to-consumer genetic testing, was confirmed as ApoE3/4. After a discussion of risks and benefits of aducanumab treatment, and associated costs, he and his family elected to initiate treatment. They agreed to the requirement for scheduled safety MRIs and the possibility of additional *ad hoc* MRIs. He and his family understood that ApoE4 carriers have a 42% risk of ARIA-E and that at best aducanumab may slow—but not improve—progressive cognitive and functional decline.

Approximately 2 weeks after the 7th aducanumab intravenous infusion (with the first maximal dose of 10 mg/kg) he developed a headache, nausea, worsening confusion, and visuospatial agnosias (partial cortical blindness). He now required assistance with basic activities of daily living including dressing, bathing, and navigating in his home. No seizures were reported. On physical examination, his mental status was markedly worse (MMSE score 12/30). His blood pressure and other vital signs were normal throughout. A brain MRI without contrast revealed amyloid related imaging abnormality-edema (ARIA-E) in posterior parieto-occipital lobes bilaterally—worse on the right—similar to the vasogenic edema found with posterior reversible encephalopathic syndrome (PRES). The ARIA-E was rated as severe (FLAIR hyperintensity >10 cm) with significant subcortical white matter and sulcal involvement—at two separate sites (both hemispheres). There were also 3 new microhemorrhages noted in the right parietal lobe (mild ARIA-H) within the area of ARIA-E. Aducanumab was discontinued due to symptomatic severe ARIA and he was admitted to the hospital for a brief course of intravenous methylprednisolone; plasmapheresis was briefly considered but not initiated. His cognitive and functional status slowly returned to baseline after 12 weeks. Monthly MRI scans revealed gradual and then complete resolution of ARIA-E at 16 weeks. Resumption of aducanumab treatment was not recommended.

This case demonstrates application of the A/T/N (Amyloid/Tau-Tangle/Neurodegeneration) biomarker classification scheme to support a clinical diagnosis of AD as well as appropriate use criteria for aducanumab treatment. This case also demonstrates the three known risk factors for ARIA with aducanumab—ApoE4 carriage, higher dose (10 mg/kg), and initial treatment period (first 8 infusions). Strategies to mitigate the risk of ARIA include excluding individuals with >4 microhemorrhages at baseline, employing a dose titration at treatment initiation, and excluding individuals taking anti-platelet or anti-coagulant drugs.

### Second-Generation Monoclonal Anti-amyloid Antibodies

With approximately 6 million individuals with AD in the US (1/3 with mild dementia) and an even greater number of older individuals with mild cognitive impairment (MCI), aducanumab treatment may be indicated for a large number of individuals in the US alone. While clinical efficacy (cognitive and functional outcomes) of aducanumab is hotly debated, the major side effects—ARIA-E and ARIA-H—are readily apparent. Although largely asymptomatic, patients may present with new neurologic signs and symptoms and require temporary or permanent suspension of treatment. As monoclonal antibodies enter the mainstream of MCI and AD management, and perhaps prevention in the future, a critical and concise review of the literature of risk factors, prevention, diagnosis, and treatment of ARIA is increasingly relevant.

Second-generation monoclonal antibodies, including aducanumab, target insoluble and fibrillar beta-amyloid (Aβ) peptides and significantly decrease CNS amyloid burden in individuals with MCI and AD—albeit with ongoing controversy regarding their clinical benefits (1). These monoclonal antibodies are emerging as potential new treatments for individuals with MCI (also known as prodromal AD) and mild dementia due to AD. The antibody treatments may also be effective in prevention of MCI and AD in cognitively normal older individuals at risk, but readouts from ongoing prevention trials of healthy at-risk individuals are pending (2023 and beyond).

Recent clinical trials of four monoclonal antibodies—aducanumab (Biogen, BIIB037), lecanemab (Eisai, BAN2401), donanemab (Lilly, LY3002813), and gantenerumab (Roche, RG1450) are generating encouraging and provocative results (Table 1). In general, these findings support the amyloid hypothesis of AD since an anti-amyloid treatment strategy also favorably alters putative downstream biomarkers of CNS tau/tangle pathology. Aducanumab is the first antibody to be FDA-approved for the treatment of MCI and AD, but not without contention. The initial approval June 7, 2021 for a broad indication (AD) was subsequently narrowed July 8, 2021 to prodromal AD (MCI) and mild dementia due to AD—restricting the indication to more closely approximate that of the study population enrolled in the pivotal phase 3 trials (7).

**Table 1:** Safety and efficacy of second-generation anti-amyloid monoclonal antibodies.

	**Aducanumab (2)**	**Lecanemab (3, 4)**	**Donanemab (5)**	**Gantenerumab (6)**
CNS amyloid clearance	+	+	+	+
Clinical stabilization	+/–	+	+	?
Tau reduction	+	+	?	+
Estimated ARIA-E incidence at the highest dose (%)	42	9.9	27.5	13.5
Estimated ARIA-H incidence at the highest dose (%)	Same as placebo	5.6	30.5	16.2

*ARIA (–E/–H), Amyloid-related imaging abnormalities (–edema/–hemorrhagic); [+] indicates that the drug is associated with the variable in the row; ? not known*.

One of the most concerning risks associated with the second-generation monoclonal antibodies is an increased risk of amyloid-related imaging abnormalities (ARIA) (8–20). ARIA is divided into two categories which may co-occur: ARIA-E and ARIA-H. ARIA-E is characterized by cerebral vasogenic edema and/or sulcal effusions/exudates best detected on T2 fluid-attenuated inversion recovery (FLAIR) MRI. ARIA-H is typically characterized as cerebral microhemorrhages and/or hemosiderosis best detected on gradient echo MRI (GRE/T2^*^), as well as less-common macro-hemorrhages and hemorrhagic stroke (21). Although ARIA may be severe in regard to new or worsened clinical signs and symptoms and brain area(s) affected, it is more typically asymptomatic. ARIA-E resolves spontaneously even with continuation of treatment in asymptomatic individuals (22). For the aducanumab studies, ARIA-E severity was based the number and size of edematous regions on MRI: a single region <5 cm was considered mild; a single region 5–10 cm or multiple regions all <10 cm, moderate; any region >10 cm, severe. For ARIA-H, a total of 1–4 new microhemorrhages was considered mild; 5–9, moderate, and >10, severe. In addition, one new area of superficial siderosis was considered mild; 2, moderate; and >2, severe.

The incidence of symptomatic ARIA is significant and problematic for clinicians treating patients with aducanumab (Aduhelm) and similar antibodies. The high incidence of drug-induced ARIA is also problematic for researchers, as evident in the donanemab phase II trial (TRAILBLAZER-ALZ) in which a large number of participants were discontinued due to ARIA (23). Several trials demonstrate that the incidence of treatment-related symptomatic ARIA is dose-dependent and this may result in differential management of patients in studies (3, 6, 15–18). The recommendation for management of symptomatic ARIA is to discontinue or reduce the drug dosage—which presents the risk of unblinding participants and researchers in clinical studies. Dosage reduction may also decrease clinical efficacy, as optimal CNS amyloid clearance requires a high dose.

#### Aducanumab

Aducanumab (Aduhelm, Biogen)—FDA-approved June 7, 2021—is a human monoclonal antibody that selectively targets aggregated Aβ (2, 14). In fact, the clone was isolated from an individual with endogenous anti-amyloid antibodies. The clinical efficacy of the drug in treating MCI and AD is debated despite its FDA approval, in part due to the premature discontinuation of the two pivotal phase III clinical trials, ENGAGE and EMERGE, following a futility analysis (24, 25). Despite the decision to terminate both trials early, both demonstrated a treatment effect in favor of aducanumab at low doses; however, the two studies were discordant at the highest dose. While the ENGAGE trial showed no treatment effect in the pre-specified primary outcome compared to placebo, the EMERGE trial showed a significant decrease in the rate of cognitive and functional decline in patients receiving high-dose aducanumab. When the two final data sets were compared to their respective futility datasets, it became apparent that the treatment effect improved in both studies as additional data were collected. The FDA cited EMERGE as “substantial evidence of effectiveness to support approval” (26). However, at best the treatment may only slow cognitive and functional decline—raising the question of what pathologies drive continued decline as amyloid burden diminishes.

In the ENGAGE trial, ARIA was the most frequent serious adverse event—occurring in 41.7% of individuals in the aducanumab 10 mg/kg group, compared to 10.2% in the placebo group. Note that these data also reveal that ARIA-E rarely occurs spontaneously (~3% in the placebo group) and may be associated with endogenous anti-amyloid antibodies (a hypothesis). ARIA-H, particularly in the form of microhemorrhages, is common in untreated older individuals, and increases with age. Most cases of ARIA are asymptomatic with the incidence of symptomatic ARIA in the 10 mg/kg group being 7.5%. Overall, the incidence of ARIA-H was lower than that of ARIA-E, and ApoE4 carriage again increased risk.

In a placebo-controlled, single-dose escalation study of aducanumab in subjects with mild-to-moderate AD, three of 53 patients developed symptomatic ARIA-E (27). All three of these individuals received the maximum dose of 60 mg/kg aducanumab and one of the three was ApoE4 homozygous. ARIA was not observed at doses below 30 mg/kg. ARIA was first observed on MRI or clinically between days 22–31 and resolved on MRI within 28 days in two cases. In the third, more severe case, ARIA-E and ARIA-H resolved in 84 days. In a study of a single patient with severe symptomatic ARIA-E and ARIA-H, symptoms were associated with malignant hypertension and epileptiform activity which resolved with intravenous methylprednisone (pulse) and levetiracetam (28). The treating clinicians note the similarity of ARIA-E with posterior reversible encephalopathy syndrome (PRES) and idiopathic cerebral amyloid angiopathy (iCAA). All three entities have a similar clinical presentation and similar MRI findings—thought to be related to CNS vascular endothelial dysfunction (21).

There is no standard of care for treatment of aducanumab-related ARIA; however, the condition is generally self-limiting and asymptomatic. Up to 65% of individuals who develop ARIA-E continue antibody treatment (19). ARIA usually appears within the first eight doses—and persists on MRI for about 4–12 weeks with self-limiting clinical symptoms lasting about 4 weeks. In other words, clinical signs and symptoms typically resolve weeks before the MRI returns to baseline. One hypothesis suggests that ARIA may be associated with increased mobilization of parenchymal amyloid to perivascular clearance pathways. Biogen investigators are collecting additional data in an ongoing open-label study of aducanumab (EMBARK) for individuals who were enrolled in prior studies. A new trial announced by Biogen will collect real-world experience with aducanumab post-approval (iCARE AD) and a Phase 4 study (mandatory for accelerated FDA-approval) will launch in 2022. In the meantime, competing anti-amyloid antibodies to treat MCI and AD are following in a similar regulatory pathway.

On January 11, 2022, the Centers for Medicare and Medicaid Services (CMS) effectively reversed FDA-approval of aducanumab by covering the cost of treatment only “with evidence-development”—in other words, only if individuals are enrolled in an approved clinical trial. Proposed studies must be designed to determine whether the treatment will result in a “statistically significant and clinically meaningful difference in cognition and function.” Clearly, biomarker evidence such as amyloid PET imaging will be considered inadequate to prove efficacy. The new guidelines also include coverage of one amyloid PET scan per lifetime to confirm amyloid positivity—essential to participate in a study of an amyloid-targeting treatment. The guidelines also limit trials to hospital-based outpatient practices and studies must meet diversity and inclusion standards to assure participation of traditionally underrepresented populations. In other words, the proposed studies must recruit and retain a population matching that of the population diagnosed with MCI and AD in the US. This will allow a more comprehensive determination of risks and benefits in all populations. The aducanumab trials, similar to most medical research and to AD research in particular, lacked diversity. This CMS decision applies to aducanumab and all other anti-amyloid antibodies for the treatment of MCI and AD.

#### Lecanemab

Lecanemab may be efficacious for individuals with AD by binding and removing soluble Aβ protofibrils (8). In the initial safety and tolerability study, the incidence of ARIA-E and ARIA-H on MRI were comparable to placebo (4). Generally, antibody treatment was well-tolerated; however, subsequent randomized double-blind trials revealed a 9.9% incidence of ARIA-E at the highest dose (10 mg/kg biweekly) (3). Similar to aducanumab, ARIA-E was found in 14.3% of ApoE4 carriers. At the request of a European regulatory authority, investigators discontinued the administration of high dose lecanemab to homozygous and heterozygous ApoE4 carriers (representing 70% of the study population), thus complicating the interpretation of results and potentially underestimating the risk or ARIA in the treated population.

#### Donanemab

Donanemab targets aggregated amyloid at an N-terminal pyroglutamate form and is therefore selective to existing amyloid deposits (29). Donanemab showed early promise by demonstrating no association with ARIA-E in a safety and tolerability study and only two cases of ARIA-H at the second highest dose (3 mg/kg, intravenous) (30). Recent phase II clinical trials demonstrated significant improvement in Integrated Alzheimer's Disease Rating Scales (iADRS) scores; however, not without a risk of ARIA. Seven individuals in the trial temporarily discontinued treatment due to ARIA and two of those seven then discontinued the study completely (5). Treatment consisted of 700 mg for the first 3 doses and 1,400 mg every 4 weeks for 72 weeks. The treatment group experienced a 26.7% incidence of ARIA with 6.1% of the treatment group experiencing symptomatic ARIA-E. ARIA-H was also greater in the treatment group. In the phase II trials of donanemab, ARIA-E occurred by week 12 and had a mean resolution time of 18 weeks. Similar to its counterparts, donanemab-induced ARIA-E was typically asymptomatic and resolved spontaneously; likewise, ARIA-E risk was higher with homozygous ApoE4 status. Interestingly, 90% of patients in the trial developed anti-drug antibodies, but their implications regarding safety and efficacy of donanemab remain unclear.

#### Gantenerumab

Gantenerumab treatment also significantly decreases CNS amyloid burden as measured by amyloid PET (31). Despite this potential, three of the five phase III trials for gantenerumab were discontinued. Two phase III trials examining the safety and efficacy of gantenerumab at higher doses (GRADUATE 1 and 2) began in 2018 and are on-going (32, 33). Application to the FDA will include results of GRADUATE 1 and 2. The open-label extensions of two clinical trials (Scarlet RoAD and Marguerite RoAD) demonstrated significant amyloid removal over 24 months in individuals with prodromal to moderate AD treated with 1,200 mg gantenerumab by subcutaneous injection every 4 weeks (31). A new trial of gantenerumab for AD prevention (SKYLINE) is in progress.

The randomized double-blind placebo-controlled phase III clinical trial (Scarlet RoAD) was halted early for futility (34). Within the study, ARIA-E incidence increased in a dose-dependent manner and was also associated with ApoE4 carriage. Investigators suggested that the optimal dosing is above 100 mg but below 330 mg given subcutaneously to maintain efficacy while minimizing risk of ARIA-E. The incidence of ARIA-E was 6.6%, occurring at a two-fold greater rate in treated patients compared to the placebo group (6). Gantenerumab-induced ARIA-E occurred typically within 3–6 months of treatment—later than its counterparts. Incidence sharply decreased after 9 months of treatment. Also similar to its counterparts, about 80% of gantenerumab-induced ARIA-E was asymptomatic and self-limiting.

## Discussion

With the FDA approval of aducanumab in June 2021, the treatment of individuals with MCI and mild AD entered into a new disease-modifying era—to be prescribed in parallel to traditional symptomatic therapies. In part to assure target engagement and decrease the risk of ARIA, appropriate use criteria for aducanumab are similar to inclusion/exclusion criteria in the phase III clinical trials. Recommended criteria are more restrictive, for example requiring evidence of CNS amyloid, than those in the package insert (7). Other anti-amyloid treatments are following a similar regulatory pathway, with anticipated accelerated FDA-approval if the standard is demonstration of CNS amyloid clearance. Other antibodies under investigation have potential advantages over aducanumab—donanemab may be the most potent, lecanemab safer, and gantenerumab administered by subcutaneous injection. These antibodies have different effects on progressive decline as shown by cognitive and functional outcomes (Table 2). The goal of treatment is slowing or halting progressive decline—compared to the placebo-treated group. While effects on CNS amyloid clearance are readily apparent, clinical outcomes are a topic of intense controversy and require further data from trials in progress (phase 3 donanemab, gantenerumab, and lecanumab) and planned (phase 4 aducanumab).

**Table 2:** Mean changes in cognitive function assessment scores from baseline compared to placebo.

**Anti-amyloid antibody**	**CDR-SB**	**MMSE**	**ADAS-Cog**	**ADCS-iADL**	**FAQ**	**iARDS**	**ADCOMS**
Aducanumab (19)	+^*^	+^*^	?	?	?	?	?
Lecanemab (3)	–	–	–	?	?	?	–*
Donanemab (5)	–	–	+	–	?	+^*^	?
Gantenerumab (105 and 225 mg) (6)	–*	–	–	?	–	?	?

*CDR-SB, Clinical Dementia Rating–Sum of Boxes; MMSE, Mini-Mental State Exam; ADAS-Cog, Alzheimer's Disease Assessment Scale-Cognitive subscale; ADCS-iADL, Alzheimer's Disease Cooperative Study–instrumental items of the Activities of Daily Living Inventory; FAQ, Functional Activities Questionnaire; iARDS, Integrated Alzheimer's Disease Rating Scale; ADCOMS, Alzheimer's Disease Composite Score*.

[+] indicates a statistically significant difference in the mean change from baseline cognitive score when compared to placebo. [–] indicates no statistically significant change in score. [?] indicates data not available. [*] values marked with an asterisk indicate the primary endpoint of the cited trial.

ARIA tends to occur within the first several weeks or months of treatment with variation depending on the drug and dosing schedule. Patients, families, and clinicians should be suspicious of ARIA if new symptoms occur within the first few treatments, particularly when the highest dose is achieved. MRI surveillance is recommended to decrease the risk of severe, symptomatic ARIA. MRI should be obtained within 12 months before treatment initiation, and before the 7th (10 mg/kg dose), and 12th infusions for aducanumab, although the appropriate use criteria recommend MRIs at the 5th (6 mg/kg), 7th (10 mg/kg), and 12th infusions, and 10th dose for ApoE4 carriers. A brain MRI should also be obtained with any new signs or symptoms suggestive of ARIA (Table 3). The need for rigorous MRI surveillance may pose additional financial and practical barrier to treatment, excluding individuals who cannot undergo MRI due to claustrophobia or with an incompatible metallic implant (such as a pacemaker).

**Table 3:** Incidence of the most common signs and symptoms of ARIA.

**Sign/Symptom**	**Aducanumab (2)**	**Lecanemab (4)**	**Donanemab (5)**	**Gantenerumab (6)**
Headache (%)	13	12.5	7.6	9.6–12.5
Dizziness (%)	4	8.3	8.4	7.7–10.4
Confusion/altered mental status (%)	5	?	?	?
Visual disturbance/eye disorders (%)	2	?	?	5.9–8.8
Nausea (%)	2	8.3	10.7	?
New onset seizure(s) (%)	?	?	?	?

*Signs/symptoms of ARIA by antibody ranked by incidence. Incidence is presented as a percentage of symptomatic patients within the total number of patients with the observation of ARIA. [?] indicates data not available*.

Risk factors for ARIA include: (1) initial treatment period, (7) higher dosage, and (8) ApoE4 genotype—with ApoE4/4 homozygotes having the highest risk (Table 4). Clinicians and patients should strongly consider ApoE genetic testing before treatment initiation as this significantly affects risk of ARIA. In fact, truly informed consent mandates ApoE genotyping. This is particularly relevant to lecanemab, which was not administered at the highest dosage to ApoE4 carriers. Risk of recurrent ARIA is exemplified in one patient with six distinct episodes of asymptomatic ARIA while being treated with aducanumab (PRIME, phase 1b) (36). The six episodes were separated in time and in brain regions affected. The ApoE4 homozygous patient had a history of hypertension. Each incidence of ARIA-E occurred shortly after a dose increase and resolved after dose reduction or suspension. ARIA-E occurred at month 7 after four 3 mg/kg doses and mild superficial siderosis (ARIA-H) occurred at month 35.

**Table 4:** Incidence of ARIA by ApoE genotype.

**Anti-amyloid antibody**	**ApoE genotype**	**Incidence of ARIA-E (%)**	**Incidence of ARIA-H/siderosis (%)**
Aducanumab (35)	ε4/ε4	64	41/33
	ε4/–	36	17/14
	–/–	20	12/6
Lecanemab (3)	ε4 positive	14.3	13.1
	ε4 negative	8.0	4.6
Donanemab (5)	ε4/ε4	44.0	
	ε4/–	30.0	19.8/17.6 (all genotypes)
	–/–	11.1	
Gantenerumab (105 mg) (6)	ε4/ε4	10.7	32.0
	ε4/-	5.4	19.8
	–/–	1.8	12.3
Gantenerumab (225 mg) (6)	ε4/ε4	?	?
	ε4/-	15.0	19.4
	–/–	11.0	11.0

*Incidence of ARIA-E and -H by ApoE genotype. ARIA-E and -H may have occurred concurrently in some individuals. [?] indicates data not available*.

Risk of ARIA and amyloid clearance both increase in a dose-dependent manner (37–39). Thus, adverse effects, amyloid clearance, and clinical benefit (slower clinical progression) may be tightly linked and result in tradeoffs regarding optimal dosage. ARIA-E may be due to saturation of pathways mediating perivascular amyloid clearance (40). The similarity of ARIA-E with other disorders of the blood brain barrier (PRES and iCAA) point to cerebrovascular dysfunction as a potential etiology. Protection of cerebrovascular integrity may reduce the risk of ARIA; likewise, the risk of ARIA may be increased in those with cerebrovascular dysfunction. There appears to be no association of ARIA with CNS amyloid burden (including regional analyses); rather, ARIA-E is often found in dependent (posterior) brain regions—similar in pattern to the vasogenic edema of PRES.

Currently, the only recommendation for treatment of symptomatic ARIA is to reduce the dose or discontinue treatment—temporarily or permanently. Treatment may continue if the patient is asymptomatic and the ARIA is mild on MRI. Radiographically moderate or severe ARIA warrants suspension of treatment. Treatment may be resumed when symptoms resolve and ARIA-E resolves on MRIs obtained every 4–6 weeks after discontinuation, but requires a frank discussion of risks and benefits. EMERGE and ENGAGE investigators continued dosing during mild ARIA-E without development of symptoms or worsening/spreading effusion (41). Reduction or suspension of dosing due to ARIA may prohibit many patients from titrating to the maximum dosage, thus limiting clinical efficacy. A standard of care for ARIA management from controlled studies is lacking, with only anecdotal evidence available to date. Current ARIA mitigation and treatment strategies with aducanumab will likely be applicable to other second-generation antibody treatments should they become FDA-approved. If symptomatic ARIA-E is presumably associated with excessive neuroinflammation, a pulse of brain-penetrant intravenous corticosteroids (dexamethasone or methylprednisolone) may be effective in minimizing severity and/or duration of symptoms. Plasmapheresis has also been attempted to manage severe ARIA-E. To date, there are no controlled studies or formal guidelines for decision-making in ARIA management and treatment.

## Conclusion

The second-generation monoclonal anti-amyloid antibodies aducanumab, lecanemab, donanemab, and gantenerumab represent a breakthrough in AD treatment and perhaps prevention. With proven CNS amyloid clearance, they have the potential to demonstrate disease-modifying clinical benefits. However, they also have significant risks of ARIA-E and ARIA-H. MRI surveillance prior to initiation of treatment, prior to dose escalations, and with any new neurologic symptom is essential. ARIA risk increases dose-dependently and is strongly associated with ApoE4 genotype, potentially limiting the optimal dose. A better understanding of the pathophysiology of ARIA-E and ARIA-H is needed to exclude those at highest risk, determine the “sweet spot” of maximal clinical efficacy with minimal risk, and optimize pharmacologic management for those individuals who develop symptomatic ARIA. Ultimately, prevention and treatment of MCI and AD with anti-amyloid antibodies may require personalized medicine—with initiation, dose escalation, and maximal dosage all adjusted to individual genotype—including but not limited to ApoE.

Questions regarding ARIA remain for the most part unanswered. How often does spontaneous asymptomatic ARIA occur? Is this associated with CNS amyloid burden? Can ARIA be predicted or diagnosed from plasma, CSF, or neuroimaging biomarkers? Does higher amyloid burden and regional localization increase ARIA risk? Which individuals are at highest risk? Will personalized medicine lower the risk of ARIA? How and why does ApoE genotype influence risk of ARIA? Can greater efficacy with anti-amyloid strategies be achieved without a concomitant higher risk of ARIA? What are the molecular and cellular pathways mediating ARIA-E and ARIA-H? What are the best treatments for individuals with moderate or severe symptomatic ARIA? Does the risk of ARIA preclude research and development of active (instead of passive) immunotherapies for MCI and AD? While studies with animal models of AD may provide novel insights (42), answers will also require further clinical research focused on these and additional new questions.

## Author Contributions

All authors listed have made a substantial, direct, and intellectual contribution to the work and approved it for publication.

## Conflict of Interest

RT is a site PI for studies of all of the monoclonal antibodies under discussion, with research funding from Biogen, Eisai, Lilly, and Roche/Genentech to Georgetown University. The remaining author declares that the research was conducted in the absence of any commercial or financial relationships that could be construed as a potential conflict of interest.

## Publisher's Note

All claims expressed in this article are solely those of the authors and do not necessarily represent those of their affiliated organizations, or those of the publisher, the editors and the reviewers. Any product that may be evaluated in this article, or claim that may be made by its manufacturer, is not guaranteed or endorsed by the publisher.
